# Ultrafast Processes Occurring in Radiolysis of Highly Concentrated Solutions of Nucleosides/Tides

**DOI:** 10.3390/ijms20194963

**Published:** 2019-10-08

**Authors:** Jun MA, Sergey A. Denisov, Amitava Adhikary, Mehran Mostafavi

**Affiliations:** 1 Department of Nuclear Science and Engineering, Nanjing University of Aeronautics and Astronautics, Nanjing 211106, China; junma@nuaa.edu.cn; 2 Collaborative Innovation Center of Radiation Medicine of Jiangsu Higher Education Institutions, Soochow University, Suzhou 215000, China; 3 Laboratoire de Chimie Physique, UMR 8000 CNRS/Université Paris-Sud, Bât. 349, 91405 Orsay, CEDEX, France; sergey.denisov@u-psud.fr; 4 Department of Chemistry, Oakland University, 146 Library Drive, Rochester, MI 48309, USA

**Keywords:** picosecond pulse radiolysis, water cation radical, hole transfer, quasi-free electron, prehydrated electron, solvated electron, transient negative ion, dissociative electron attachment

## Abstract

Among the radicals (hydroxyl radical (^•^OH), hydrogen atom (H^•^), and solvated electron (e_sol_^−^)) that are generated via water radiolysis, ^•^OH has been shown to be the main transient species responsible for radiation damage to DNA via the indirect effect. Reactions of these radicals with DNA-model systems (bases, nucleosides, nucleotides, polynucleotides of defined sequences, single stranded (ss) and double stranded (ds) highly polymeric DNA, nucleohistones) were extensively investigated. The timescale of the reactions of these radicals with DNA-models range from nanoseconds (ns) to microseconds (µs) at ambient temperature and are controlled by diffusion or activation. However, those studies carried out in dilute solutions that model radiation damage to DNA via indirect action do not turn out to be valid in dense biological medium, where solute and water molecules are in close contact (e.g., in cellular environment). In that case, the initial species formed from water radiolysis are two radicals that are ultrashort-lived and charged: the water cation radical (H_2_O^•+^) and prethermalized electron. These species are captured by target biomolecules (e.g., DNA, proteins, etc.) in competition with their inherent pathways of proton transfer and relaxation occurring in less than 1 picosecond. In addition, the direct-type effects of radiation, i.e., ionization of macromolecule plus excitations proximate to ionizations, become important. The holes (i.e., unpaired spin or cation radical sites) created by ionization undergo fast spin transfer across DNA subunits. The exploration of the above-mentioned ultrafast processes is crucial to elucidate our understanding of the mechanisms that are involved in causing DNA damage via direct-type effects of radiation. Only recently, investigations of these ultrafast processes have been attempted by studying concentrated solutions of nucleosides/tides under ambient conditions. Recent advancements of laser-driven picosecond electron accelerators have provided an opportunity to address some long-term puzzling questions in the context of direct-type and indirect effects of DNA damage. In this review, we have presented key findings that are important to elucidate mechanisms of complex processes including excess electron-mediated bond breakage and hole transfer, occurring at the single nucleoside/tide level.

## 1. Introduction

This review begins with a summary on the earliest processes of water radiolysis highlighting that in dilute solutions only the indirect effect of radiation is predominant and neither direct ionization (direct effect) nor the reactions of the water cation radical (H_2_O^•+^) and presolvated electron are important (Section 1.1). Recently, a comprehensive picosecond (ps) pulse radiolysis measurements in highly concentrated aqueous solutions of inorganic acids and of salts showed the importance of the direct ionization of the solute in addition to the primary charge transfer from H_2_O^•+^ and the presolvated electron (Section 1.2). To study the physico–chemical processes that are involved in the radiation damage to the biologically important macromolecules, time-resolved studies using ps pulse radiolysis were employed in concentrated solutions of monomeric DNA/RNA model systems (Section 1.3). Studies on ultrafast hole (backbone-to-base, base-to-backbone, and phosphate-to-sugar) transfer were reviewed (Section 2.1). Additionally, studies including time-resolved investigations (e.g., femtosecond pump-probe laser spectroscopy, ps pulse radiolysis) on electron transfer processes (Section 2.2 to Section 2.2.4) including radiation-produced electron-mediated bond dissociation in nucleosides were reviewed. This review summarizes the results of these ps pulse radiolysis experiments and their implications to understand the mechanisms involved in radiation damage to DNA and to radiotherapy (see conclusion).

### 1.1. Ultrafast Processes in Water Radiolysis

The primary species formed due to ionization of water molecule by highly energetic photons or charged particles is the water cation radical, H_2_O^•+^. Via subsequent ultrafast proton transfer, H_2_O^•+^ produces H_3_O^+^ and ^•^OH (Reaction R1) [1,2,3]. On the other hand, the ejected secondary electrons (SEs) with an estimated quantity of ~4 × 10^4^ electrons per 1 MeV energy deposited, can cause cascades of additional ionizations and excitations by inelastic scattering through coulombic interactions with solvent molecules [4,5,6]. As a result, low-energy electrons (LEEs) are generated with an excess kinetic energy of 0–20 eV, named quasi-free electron (e_qf_^−^) [1,2,3,4,5,6,7,8], and successively lose their kinetic energy to become thermalized (e_th_^−^) with a time constant of less than tens of femtoseconds (fs) in water under ambient conditions [4,9]. Further, these electrons can either recombine with H_2_O^•+^ (Reaction R2) or undergo multistep solvation prior to their complete localization as the solvated electron, e_sol_^−^ (Reaction R3) [4,7,8,9]. The multistep solvation of e_qf_^−^ is completed within 1 ps in water and extended to tens of picoseconds in alcohols [4,7,9,10,11,12,13,14,15,16,17,18,19,20,21]. The characteristic timescale of proton transfer along the hydrogen bond from H_2_O^•+^ to the H_2_O molecule in liquid water is 30–200 fs [9,15]. In addition, dissociation of the excited state of water (H_2_O*) leads to ^•^OH and H-atom (G = 0.5 mol J^−1^) via molecular fragmentation (Reaction R4) at electronic transition timescale. The reactions (R1–R4) proceed faster than the diffusion rate of radicals along the track and these are often thought to be the earliest physicochemical events in water radiolysis [22,23,24,25,26].

(R1) $$H_{2}O^{•+}+H_{2}O\to {}^{•}\mathrm{OH}+H_{3}O^{+}$$

(R2) $$H_{2}O^{•+}+e^{-}\to H_{2}O^{*}$$

(R3) $$e_{\mathrm{qf}}^{-}\;\left(E_{c}<20\;\mathrm{eV}\right)\;\to e_{\mathrm{th}}^{-}\;\left(E_{c}=0.025\;\mathrm{eV}\right)\;\to e_{\mathrm{pre}}^{-}\to e_{\mathrm{sol}}^{-}$$

(R4) $$H_{2}O^{*}\to {}^{•}\mathrm{OH}+H^{•}$$

Knowledge of the initial and primary radiolytic yields of each water-derived radical at room temperature is the key issue in fundamental and applied radiation chemistry. Note that the “initial” yields are the yields of the species at the physicochemical stage of radiation events (~1 ps) and the “primary” yields are yields of the species at the chemical stage (at 200 ns) [22,24,25,26]. Now, thanks to the development of ps pulse radiolysis, radiolytic yields of the species, such as the hydrated electron (e_sol_^−^) and ^•^OH at a few picoseconds, have been reported recently [27,28].

In dilute solutions (i.e., at concentrations, ca. 10^−3^ M or lower), the direct ionization of the solute itself is negligible because most of the radiation energy is absorbed by the solvent [1,2,3,29]. The charge transfer processes that involve radiation-produced excess electron transfer due to the ionization of the solvent to the solute molecule as well as hole (i.e., unpaired spin) transfer from H_2_O^•+^ to the solute molecule is not likely to take place at a long distance [29,30,31]. As a result, the ultrafast processes in dilute solution are identical with those in neat water (Reactions R1 to R4) and the dominant reaction pattern was established by the quasi-diffusion controlled reactivity of secondary water-derived radicals ^•^OH, H^•^, and e_sol_^−^ [29,30,31,32]. On the other hand, at concentrations ca. 0.1 M or higher, the processes due to direct ionization of the solute (direct effect) along with the charge transfer processes from the surrounding solvent-derived radicals (e.g., H_2_O^•+^ from H_2_O) to the solute become non-negligible [29,30,31].

### 1.2. Effect of Radiation in Concentrated Solutions

Many applications of ionizing radiation deal with concentrated solutions. For example, the spent nuclear fuel reprocessing technology utilizes highly concentrated nitric acid solutions [33]. Recent wastewater treatment via electron-beam techniques requires extensive studies in concentrated aqueous solutions. To elucidate these processes, it is necessary to know the mechanisms involved in the radiation-mediated formation of radicals, and their yields in highly concentrated nitric acid solutions, (e.g., nitrate radical (NO_3_^•^), nitrite radical (NO_2_^•^) [33,34,35]) as well as some elementary reactions between organics and water radicals in complex systems.

The concentrated solutions could also be employed as model systems to investigate radical reactions that are observed in the interfacial and biological systems [29,30,31]. Note that such reactions are not easy to study directly by the pulse radiolysis technique. The global concentration of macromolecules (DNA, RNA, proteins, etc.) in the cell nucleus are in the range of 65–220 mg/mL; therefore, the cell nucleus is not a homogeneous dilute aqueous solution [36]. Furthermore, dsDNA in a cell is densely packaged in nucleosomes, with the DNA wrapped around the histone protein core that forms the basic unit of the chromatin structure [36,37]. When ionizing radiation is applied, a part of the radiation energy is absorbed directly by biomolecules (direct effect), but a part is also absorbed by the water layer adjacent to biomolecules [31]. Therefore, the extent of contributions of the ultrafast effect and the indirect effect on the damage are complex and very difficult to understand if only observations in dilute solution are employed as the reference for such systems [29,30,31]. For instance, one-electron oxidants (SO_4_^−•^, Cl_2_^−•^, etc.) in dilute solutions or electron spin resonance (ESR) spectroscopy of irradiated samples in homogeneous frozen aqueous glassy solutions have been generally used to model the pathways involved in direct-type effects of radiation; however, these experiments do not exactly model the cellular environment at ambient temperature [30,31,38,39].

**Figure 1:**
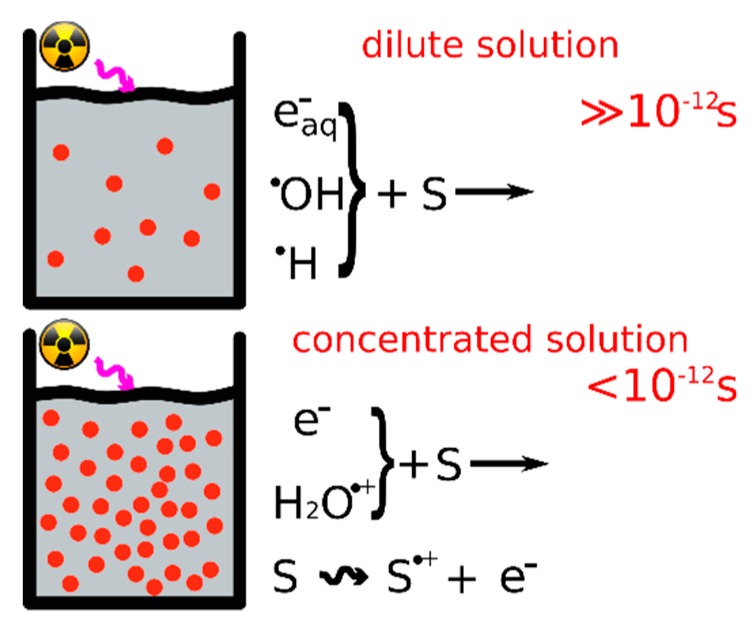
Ultrafast charge transfer from the water cation radical, H_2_O^•+^, and excess electron attachment to the solute occurring in less than 1 ps in concentrated solutions; these processes do not occur in dilute solutions and reactions due to indirect effect of radiation are predominant in dilute solutions.

While studying concentrated solutions of halides, a possible reactivity of the excess electron and H_2_O^•+^ has been evoked by Hamill et al. [40]. This hypothesis remained under debate for several decades due to the limitation of the time resolution and to not having adequate knowledge of the yield of e_sol_^−^ and ^•^OH in the picosecond range. In the past decade, as represented in Figure 1, the electron transfer from solutes to the primary positive holes from the solvent in aqueous systems as an ultrafast pathway has been demonstrated quantitatively in solutions containing a large amount of halide salts and in acidic solutions (HNO_3_, H_2_SO_4_, H_3_PO_4_, etc.) [1,2,3,34,41]. The recently developed laser-driven picosecond pulse radiolysis technique has been established as an exciting new capability that provides > 5 ps time resolution for probing the dynamics of transient species and extends the capability to investigate chemical reactions covering a broader time range [42]. Even though the direct time resolution of our technique is limited to > 5 ps that cannot probe the dynamics exactly in real time, measurement of secondary radical formation yields allows us to address quasi-directly the reactivity of H_2_O^•+^ and presolvated electrons by altering the solute concentration (Figure 1).

Applying this above-mentioned technique, we have shown that three distinct processes are involved in the formation of secondary radicals such as phosphate and nitrate radicals [35,41]: (i) reaction of ^•^OH with phosphate anions and undissociated H_3_PO_4_ and HNO_3_ (indirect effect), (ii) direct ionization of phosphoric acid (direct effect of ionizing radiation on solutes), and (iii) reaction of H_2_PO_4_^−^and NO_3_^−^ with H_2_O^•+^. On the other hand, by observing a decreasing initial yield of e_sol_^−^ as a function of H_3_O^+^ concentration at a similar timescale, we showed that the radiation-produced excess electrons reacted rapidly with H_3_O^+^ [41]. Our results further established that the rate of this reaction is faster than the corresponding reaction of e_sol_^−^ by two orders of magnitude [41]. Thus, our results highlight the difference of the decay of a variety of water-derived radicals in the dilute and concentrated medium, and this is schematically represented in Figure 1.

### 1.3. Studies of Biomolecule Model Systems

**Figure 2:**
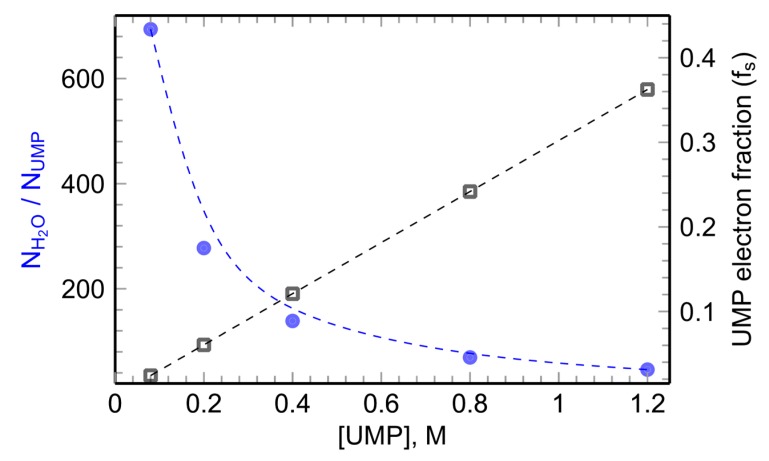
Left axis (blue filled circle): molecular number ratio between water and UMP plotted as a function of UMP concentration in an aqueous environment. Right axis (open box): correlation of the electron fraction (*f_s_*) with the UMP concentration to indicate the probability of direct-type radiation effect on the nucleotide occurring in the bulk phase. Reprinted with permission from [43]. Copyright, 2018, American Chemical Society.

Radiolytic studies of biomolecules (e.g., DNA) are more challenging compared to inorganic systems because of their structural complexities. This can be illustrated as follows: at first, the estimation of the extent of direct ionization of the solute and H_2_O^•+^ mediated oxidation of the same solute in bulk solutions is not well-established. For instance, in aqueous solutions of 5′-uridine monophosphate (UMP or URP (Uracil base (U), Ribose(R), and Phosphate (P))), correlation of the electron fraction (*f_s_*, Section 2.1.1) of UMP with UMP concentration indicates the probability of the direct effect (or, direct ionization) on the nucleotide occurring in the bulk phase, while a few of their corresponding cation or anion radicals were quantitatively measured at ambient conditions (Figure 2).

Secondly, in contrast to the small inorganic cation radicals, ultrafast formation of a DNA-hole i.e., a DNA cation radical ((DNA)^•+^) occurs upon ionization, resulting in the formation of holes with unpaired spins on the bases and on the sugar–phosphate [30,31,38,39,44,45,46]. A substantial volume of work has been focused on radiation-induced hole-mediated damage in DNA via the oxidation pathway, as this pathway may lead to cellular death [29,30,31,38,39,43,44,45,46,47,48,49,50,51,52,53,54,55,56,57,58,59,60,61,62,63,64,65,66,67,68]. A fast base-to-base hole transfer process [29,30,31,38,39,43,44,45,46,47,48,49,50,51,52,53,54,55,56,57,58,59,60,61,62,63,64,65,66,67,68,69,70,71,72] and backbone-to-base hole transfer process [30,31,38,39,43,44,45,46] leads to the localization of these holes at a nearby guanine base. It has been well-established that the stacks of guanine, for example, GG, GGG, etc. are the most stable hole localization sites after long range hole transfer [29,30,31,38,39,43,44,45,46,47,48,49,50,51,52,53,54,55,56,57,58,59,60,61,62,63,64,65,66,67,68,69,70,71,72]. Previous laser spectroscopic measurements or the use of one-electron oxidants (SO_4_^•−^), which had often been used to model the pathways involved in DNA damage via the direct effect, were not able to probe these dynamics immediately following the ionizing radiation [29,30,31,38,39,43,44,45,46,47,48,49,50,51,52,53,54,55,56,57,58,59,60,61,62,63,64,65,66,67,68,69,70,71,72]. Another important point is that the dynamics of transfer and the localization of the radiation-produced excess electrons in DNA in the liquid phase are not fully understood [4,8,29,30,31,38,39,56,57,58,59,60,61,62,73,74,75]. When excess electron attachment occurs on a nucleoside target, it leads to a variety of excited states of the anion radicals, which can either undergo bond dissociation or relaxation to their ground states [4,73,74,75]. Therefore, the extent and pathway of the radiation-produced excess electron-mediated reactions in bulk solutions are important and certainly play a key role in radiation therapy and radiation protection.

## 2. Ultrafast Hole and Electron Transfer under Irradiation

### 2.1. Elucidation of Pathways of Hole Transfer Processes Employing Concentrated Nucleotide Solutions

#### 2.1.1. Backbone-to-Base Hole Transfer

**Scheme 1:**
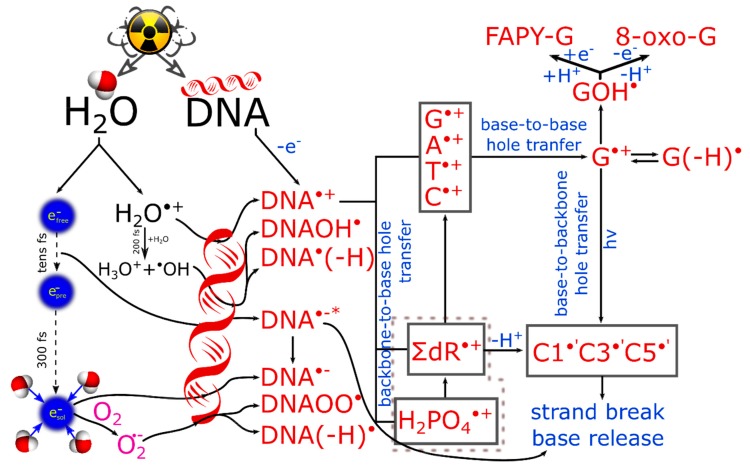
This scheme represents the important reactions involved in direct-type and in indirect effects of radiation leading to DNA damage. After formation of the “hole” (i.e., the unpaired spin) via direct-type effect, the hole is localized on the guanine base via base-to-base and backbone-to-base hole transfer processes [30,31,38,39,43,44,45,46,66]. Reactions of the localized hole on the guanine base in DNA are schematically shown as well [29,30,31,43,47,48,49,50,51,52,53,54,55,56,57,58,59,60,61,62,63,64,65,66,69]. For successful formation of a neutral sugar radical, a rapid deprotonation must occur from the one-electron oxidized sugar–phosphate backbone before a competitive backbone-to-base hole transfer takes place [30,31,43,45,46]. Sugar radicals are precursors of DNA-strand breaks [29,30,31]. The competition between the solvation of radiation-produced electrons and their reactions with DNA leading to formation of strand break via DNA^•−^* [4,8,29,30,31,45,73,74,75] are mentioned. In addition, formation of superoxide anion radical (O_2_^•−^) and its reactions [29] are also shown.

In irradiated DNA, holes localize on the most electropositive base, guanine, through direct ionization and base-to-base and backbone-to-base hole transfer processes (Scheme 1) [30,31,38,39,44,45,46,66,76,77]. The best overall estimate of the probability of direct ionization at a given site in DNA, such as the sugar, phosphate, or DNA base is provided by the number of valence electrons at that site (electron fraction (*f_s_* = no. of valence electrons at that site/total no. of electrons at that site) (Figure 2)) [30,31,38,39,44,45,46]. For DNA, ca. 43% of the ionizations should initially occur at the bases and the remainder at the sugar-phosphate moiety [30,31,38,39,44,45,46]. These are valence electron ionization events. However, electron spin resonance (ESR) studies of trapped DNA-radicals at 77 K [30,31,38,39,44,45,46] and damaged base release studies of irradiated hydrated DNA at room temperature [78] show that backbone-to-base hole transfer process increases the extent of trapped holes on the bases to ca. 77% (Scheme 1) mainly on the guanine base. Based upon the electron density/fraction of the phosphate group, it is expected that phosphate radicals formed via ionization events in the DNA-backbone must play an important role in the backbone-to-base hole transfer process [30,31,38,39,43,44,45,46]. However, earlier ESR studies at 77 K on gamma-irradiated (77 K) hydrated (Γ = number of water molecules/nucleotide = 12 ± 2) DNA [30,31,38,39,45], on X-irradiated (77 K) DNA-models in frozen aqueous solution [30,31,45], on X-irradiated single crystals of alkyl phosphates, organic phosphates, and sugar–phosphates [30,31,45,79,80,81,82], and on gamma-irradiated (77 K) dimethyl phosphate in both frozen aqueous solution and neat [45], showed the formation of carbon-centered radicals and not of phosphate radicals. We also note here that ESR spectra of phosphate radicals were reported from an X-ray irradiated (77 K) single crystal of 1,2-*o*-isopropylidene-3,5-*o*-phenoxyphosphoryl-α-D-xylofuranose [83]. The oxyl radicals, such as the phosphate radicals (PO_3_^•2−^ (E^0^/V (standard electrode potential, PO_3_^•2−^ + H^+^ + e^−^ = HPO_3_^2−^) = 1.54), H_2_PO_4_^•^ (E^0^/V = 2.75 [84]) have been shown to be strong one-electron oxidants. In addition, laser flash photolysis studies of a ribose-5-phosphate solution at room temperature have provided evidence of fast (5 × 10^7^ s^−1^) intramolecular H-atom abstraction by phosphate radical (O_3_PO^•^) [85].

It is evident that the backbone-to-base hole transfer process should occur by very short-lived phosphate radical-mediated oxidation of the bases followed by a base-to-base hole transfer process (Scheme 1). Consequently, to provide evidence of the phosphate radical mediated oxidation of bases, which is the key step to model the backbone-to-base hole transfer process (Scheme 1), picosecond pulse radiolysis studies of the reactions between H_2_PO_4_ and the DNA bases- G, A, T, and C in 6 M H_3_PO_4_ at room temperature were performed [44].

**Figure 3:**
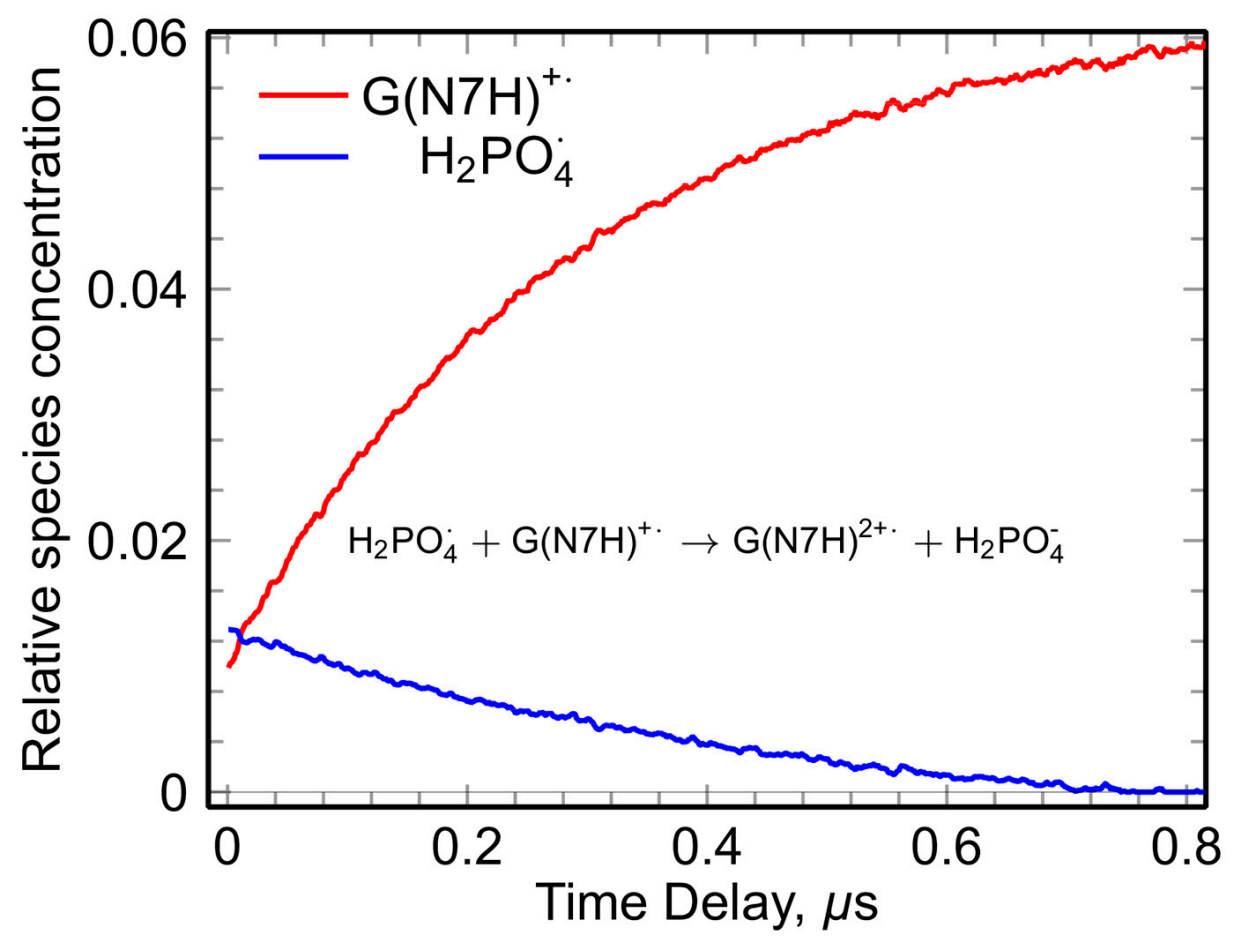
The intermolecular hole transfer reaction between protonated guanine and H_2_PO_4_˙ formed by direct ionization and H_2_O^•+^ mediated oxidation in 6 M H_3_PO_4_ [44]. Reprinted with permission from [44]. Copyright, 2018, Royal Society of Chemistry.

The phosphate radical, H_2_PO_4_^•^, was generated by direct ionization and via H_2_O^•+^ mediated oxidation of H_2_PO_4_^−^ followed by deprotonation in 6 M H_3_PO_4_ [44]. Analyses of the UV-visible pulse radiolysis spectra provided evidence for formation of DNA-base radicals via direct one-electron oxidation of individual DNA bases (G, A, and T) by H_2_PO_4_^•^ in 6 M H_3_PO_4_ (Figure 3 and Table 1). However, the rate of oxidation of protonated cytosine by H_2_PO_4_^•^ appeared to be too slow to detect. This work showed that H_2_PO_4_^•^ oxidizes nucleobases bimolecularly. These results led to the conclusion that H_2_PO_4_^•^ formed via direct ionization events in the sugar-phosphate backbone would oxidize the DNA bases; thus, these results could be treated as benchmarks of the backbone-to-base hole transfer process (Scheme 1). These results point out that the formation of the doubly charged DNA base cation radical (e.g., G^2+•^) from the activated complex (e.g., G(N7H^+^)—H_2_PO_4_^•^) is much faster than the diffusion of reactants (i.e., G(N7H^+^) and H_2_PO_4_^•^) and is mediated by single electron transfer [44].

Of course, this particular pulse radiolysis study is limited to the fundamental model that represents only the reactions between phosphate radicals and DNA bases in a highly concentrated medium (6 M H_3_PO_4_). However, by taking into account a 3-dimensional model of charge transfer processes in DNA [86] and the proximity of the phosphate group to the base, the corresponding reaction in DNA could occur either by intramolecular through-bond electron transfer, or via intermolecular through-space electron transfer [44]. Therefore, the parameters that govern these two reactions (i.e., intermolecular and intramolecular) should be different. Nevertheless, this ps pulse radiolysis work demonstrated the direct observation of oxidation of the weakly protonated base, thymine by H_2_PO_4_^•^, and can be treated as a model of backbone-to-base hole transfer [44].

#### 2.1.2. Base-to-Backbone and Phosphate-to-Sugar Hole Transfer Process

ESR studies of irradiated DNA and irradiated model systems at 77 K showed that for successful formation of a sugar radical, a rapid deprotonation must occur from the one-electron oxidized sugar-phosphate backbone before a competitive backbone-to-base hole transfer take place [30,31,43,45,46]. As a result, the picosecond pulse radiolysis measurements were extended to study the reactions of phosphate radicals with various concentrations of monomeric DNA or RNA-models, i.e., uridine 5′-monophosphate (UMP), uridine (Urd), uracil (U), ribose (Rib), and phosphate (P), that are biologically relevant [43]. Uracil derivatives were preferred to other nucleotides/sides because of their unique solubility in water. The radiation-mediated direct ionization of the sugar-phosphate moiety in UMP as well as the interaction of the sugar–phosphate moiety in UMP with H_2_O^•+^ become experimentally observable at ambient temperature and in solutions having high concentrations of UMP, which is associated with higher electron fraction.

In highly concentrated UMP aqueous solutions [43] (> 0.2 M) and just after the electron pulse, an absorption band was found to develop with a maximum at 520 nm. This band was attributed to the phosphate radical (H_2_PO_4_^•^) that was formed either by direct ionization or by electron transfer to H_2_O^•+^. This assignment was based on the fact that the radical intermediate at 520 nm was found to be present only in UMP and was not detected either in the nucleoside (Urd) or in the base (U). The shape of this band was found to be nearly identical to that of the phosphate radical (H_2_PO_4_^•^) previously found in the pulse radiolysis of concentrated phosphoric acid [1,2,3,41,44]. Additionally, the cation radical of ribose absorbs in the UV range below 300 nm, and the uracil base cation radical (U^•+^) from U has a different spectral shape. Thus, the possibilities that the location of the observed cation radical was either at the sugar or at the base were ruled out [43].

**Scheme 2:**
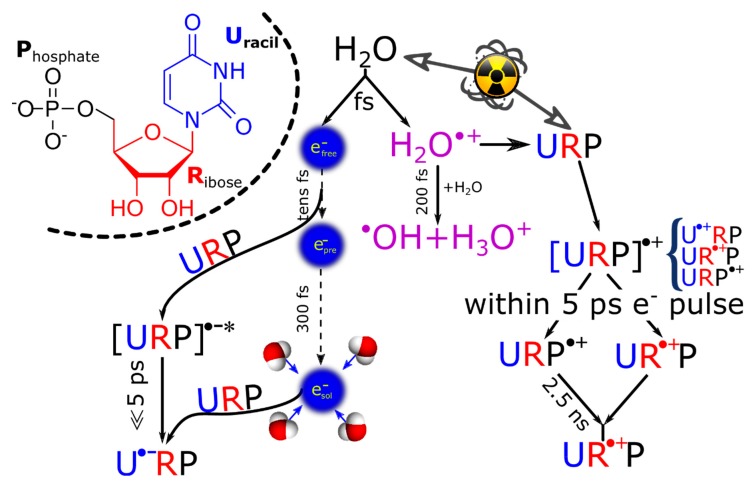
Ultrafast processes of charge transfer occurring in radiolysis of highly concentrated UMP water solutions. URP represents the composition of uracil base (U), ribose (R), and phosphate (P). Adapted with permission from [43]. Copyright, 2018, American Chemical Society.

Due to the lower redox potential of the uracil base moiety relative to those of sugar and phosphate moieties [29], the holes are initially trapped on the uracil base in UMP, i.e., formation of U^•+^MP occurs at first [43]. However, a facile and subsequent base-to-sugar hole-transfer process happens in a conformation of UMP^•+^ in which the sugar–phosphate moiety is proximate to U^•+^MP (Scheme 2). This ultrafast process of hole transfer to ribose occurs at a shorter time than the ≤ 7 ps time resolution of our pulse radiolysis measurements and was not observed. At a higher concentration of Urd (1.5 M) no transient signal of U^•+^rd was detected either [43]. The absence of signals due to U^•+^MP and (Urd)^•+^ absorptions led to the conclusion that an intramolecular charge transfer process from base-to-sugar occurred within a picosecond time window in U^•+^MP and [Urd]^•+^. This base-to-sugar hole transfer might occur via tunneling and is similar to those found in one-electron oxidized gemcitabine [87,88] or migrate as was found for a phosphate system in water (Scheme 2) [43]. The directly formed UR^•+^P (Scheme 2) was not detected due to the high concentration issues (the transient species could not be observed due to the high absorption of the unreacted solute in the UV region) [43,44]. Moreover, pulse radiolysis experiments with ribose-5-phosphate (RP) did not succeed because RP was found to rapidly degrade under radiation [43].

**Figure 4:**
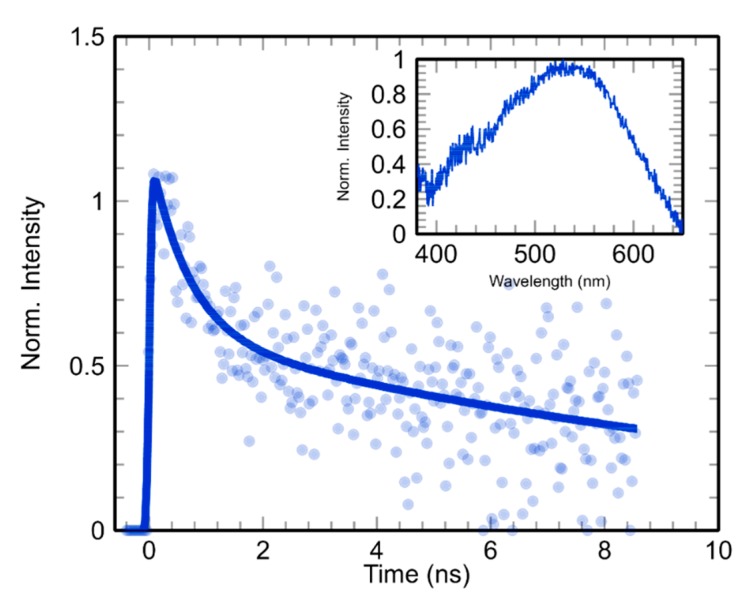
The intramolecular phosphate-to-sugar hole transfer kinetics and (inset) relevant spectral observation of phosphate radicals in aqueous nucleoside solutions upon ionizing radiation. The blue circles are experimental data points and the solid line is the best fit of these points.

The hole localized on the phosphate subunit of UMP (UMP^•+^), at very short time, was shown to undergo a nearly first-order decay with a time constant of ca. 2.5 ns (Figure 4). This was assigned to a phosphate-to-sugar hole transfer process. We note here that the hole transfer process is likely to be either intramolecular or intermolecular [89]. Based on a model study of the bimolecular reaction between H_2_PO_4_^•^ and ribose under highly acidic conditions in H_3_PO_4_ solutions at longer time scale presenting evidence of a similar reaction (H_2_PO_4_^•^ + Ribose → Ribose^•+^ + H_3_PO_4_), the intermolecular hole transfer pathway was ruled out [66]. These results clearly showed that the Rib unit of UMP repairs P^•+^ intramolecularly in a few ns (Scheme 2) [43].

This phosphate-to-sugar hole transfer process is not temperature-activated as no noticeable impact on the lifetime and decay rate of UMP^•+^ (URP^•+^) was observed up to 60 °C. This experimental result showed that the activation barrier of this hole-transfer process was negligible [43]. These results clearly establish that the mechanism of formation of neutral sugar radicals is dominated by the fast deprotonation of sugar cation radicals, which form through a very rapid base-to-sugar hole transfer or phosphate-to-sugar hole transfer in the ground state (Scheme 2) [30,31,38,39,43,44,45,46,66]. In addition, sugar radical formation has been observed via a rapid charge and spin transfer process from base to the sugar in the excited state of purine and pyrimidine base cation radicals (Scheme 1) [30,31,38,39,45,90,91,92,93,94,95]. Hence, the decay of UMP^•+^ observed in UMP as well as the decay of H_2_PO_4_^•^ in H_3_PO_4_/ribose solutions provides evidence for a rapid phosphate-to-sugar hole-transfer process and subsequent deprotonation of the sugar cation radical thus formed. Accounting for the phosphate-to-sugar hole-transfer process, we consider that once formed, UR^•+^P remains stable for tens of nanoseconds in H_3_PO_4_/ribose solutions. It is well-established in the literature that carbon-centered neutral radicals formed in the 2′-deoxyribose moiety of the sugar–phosphate backbone are precursors of DNA-strand breaks [29,30,31,91,92]. Therefore, tracking the sugar radical formation in DNA is of crucial importance to elucidating the biological consequences of radiation [91,92].

### 2.2. Excess Electron-Mediated Bond Dissociation in Bulk Solutions

**Scheme 3:**
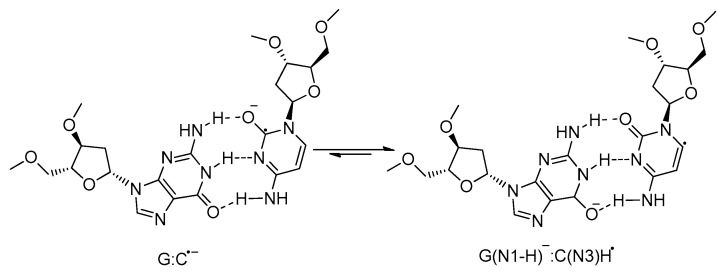
Reversible protonation at N3 of C^•−^ from N1 of the complementary guanine base leading to the formation of G(N1–H)^−^:C(N3)H^•^.

Although works from the late 70′s to date have established that radiation-produced electrons in their various stages exhibit different reactivity towards DNA subunits-bases and the sugar-phosphate backbone—this field is hence under active debate [4,8,29,30,31,38,39,45,56,57,58,59,60,61,62,63,64,70,71,72,73,74,75,88,96,97,98,99,100,101,102,103,104,105,106,107,108,109,110,111,112,113]. The consensus that emerged from extensive studies by various groups are the following:

a. The radiation-produced excess electrons are trapped on the DNA-bases upon thermalization.

b. Similar to the base-to-base hole transfer processes (Scheme 1 and Section 2.1), via tunneling, the radiation-produced excess electrons that are attached to all bases and the backbone, are trapped by the most electron-affinic bases (T and C) resulting in the formation of thymine and cytosine anion radicals (T^•−^ and C^•−^, Scheme 1) so that guanine and adenine anion radicals (G^•−^ and A^•−^) are not observed even at 4 K by ESR spectroscopy [29,30,31,38,39,45,56,57,58,59,88].

c. The base anion radicals, being stronger Brönsted bases than their parent compounds, have been shown to undergo both reversible and irreversible protonation reactions [29,30,31,38,39,45,56,57,58,59,88]. Four K ESR/ENDOR studies of X-ray irradiated single crystals of cytosine monohydrate have provided evidence of reversible protonation of C^•−^ at N3 [29,30,31,56,57,58,59,88] leading to C(N3H)^•^. In addition, C(N3H)^•^ formation in DNA (Scheme 3) via reversible protonation at N3 of C^•−^ from N1 of the complementary guanine base has been reported [29,30,31,38,39,45,56,57,58,59,60,61,62,64,88,114]. Due to the above-mentioned reversible protonation of C^•−^ at N3 in G:C base pairs, the efficiency (rate and extent) of excess electron transfer through base-to-base in dsDNA is decreased but not completely stopped [29,30,31,55,56,57,58,59]. However, T^•−^ in dsDNA does not undergo similar reversible protonation and the efficiency of excess electron transfer is not affected in A:T base pairs [29,30,31,57,58,59]. ESR studies employing gamma-irradiated (irradiation was carried out at 77 K) hydrated (Γ = 12 ± 2 water molecules per nucleotide) DNA showed that the T^•−^ population decreased from ca. 50% to 25% with a simultaneous increase in C(N3H)^•^ population as the temperature increased from 77 K to 130 K. After progressive increases of temperature at and above ca. 160 to 170 K, T^•−^ has been shown to undergo irreversible protonation from the surrounding solvent (H_2_O) to form the C6-hydrogen adduct radical, TH^•^, the earliest characterized and the most well-researched radical employing ESR spectroscopic studies of irradiated DNA [30,115].

d. The various stages of radiation-produced electrons leading to its complete solvation (Reaction R3 and Scheme 1) have been extensively studied using various techniques, e.g., pump-probe femtosecond laser spectroscopy, picosecond pulse radiolysis, ESR, and theory [7,8,9,15,22,23,24,25,26,28,30,31,70,71,72,73,74,75,88,111,112,113,116,117,118]. e_sol_^‒^ do not cause cleavage at the sugar-phosphate backbone leading to strand breaks [29,30,31,88,111,112,113]. Rather, these electrons either reside on biomolecules as anion radicals that undergo subsequent electron transfer, or they undergo irreversible protonation from the surrounding solvent leading to hydrogen-adduct radicals [29,119]. However, in 1984, Loman and his group were the first to report that “dry electrons” formed in gamma-irradiated frozen anoxic solutions of DNA caused strand breaks [110]. This study remained obscure until 2000 and onwards, at which time well-defined electron beam experiments carried out by Sanche and various other groups discovered that LEEs caused single and double strand breaks (SSBs and DSBs), both in DNA-model systems in gas and in condensed phases through dissociative electron attachment (DEA); these studies have been extensively reviewed [4,8,29,30,31,38,39,43,45,57,58,59,73,74,75,96,97,98,99,100,101,102,103,104,105,106,107,108,109,120,121].

**Scheme 4:**
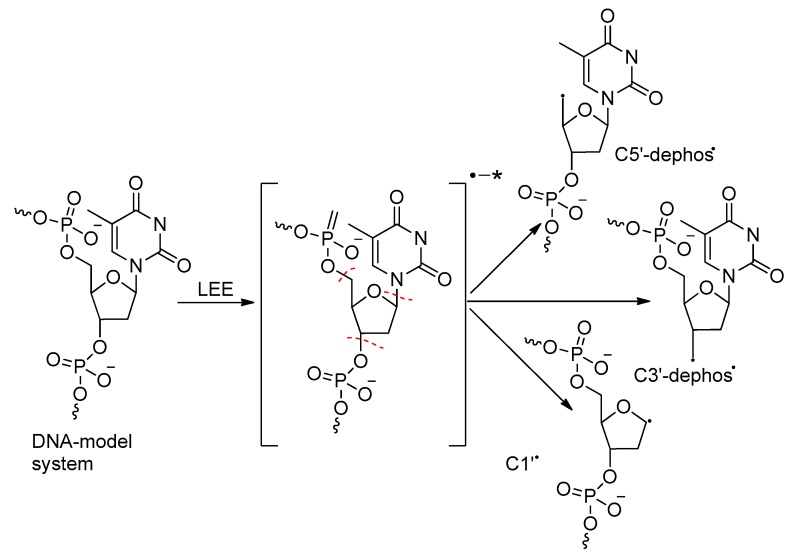
LEE-induced predominant sugar–phosphate cleavage pathways in DNA-model systems.

The Sanche group showed that LEEs below ca. 4 eV can induce SSBs in plasmid DNA via DEA. Recently, the Sanche group has provided evidence that LEEs (0.5 to 30 eV) can lead to clustered lesions [102]. Extensive experimental and theoretical studies have established that the initial step involves electron capture into the unoccupied molecular orbitals that are above the lowest unoccupied molecular orbitals (LUMOs) of the parent nucleobase. This is termed a “shape resonance”, thereby creating excited transient negative ion (TNIs*). Note that these “shape resonances” are well-characterized in the gas phase, but they collapse in condensed phases [4,8,98]. The TNIs*, after being formed, could rapidly lead to direct (or, frank) strand breaks through sugar-phosphate (C3′–O3′ or C5′–O5′) σ bond cleavage or result in unaltered base release via N1–C1′ glycosidic bond breakage (Scheme 4) [4,8,29,30,31,38,39,43,45,57,58,59,73,74,75,96,97,98,99,100,101,102,103,104,105,106,107,108,109,120,121]. These experiments were carried out either in vacuum or in microhydrated molecular targets that cannot account for the conditions in living cells. In addition, experimental studies on cross sections of samples irradiated by LEEs including product analyses of these samples coupled with theoretical studies have shown that DEA channels are influenced by N-atoms of the base [122,123].

#### 2.2.1. Can Prehydrated Electrons (e_pre_^−^) Cause Bond Breakage Via DEA?

In irradiated homogeneous aqueous LiCl glasses, radiation-produced electrons exist in shallow traps of ca. −0.5 eV and hence, these electrons are considered to be e_pre_^‒^ [30,120,121,122,123,124,125,126,127,128,129]. Some 77 K ESR studies of reactions of e_pre_^−^ with N-acetylalanylalanine methyl ester, N-acetylproline, and glycine methyl ester along with theoretical calculations employing density functional theory (DFT) provide evidence of bond breakage via DEA [124,125]. For example, addition of radiation-produced e_pre_^‒^ to N-acetylalanylalanine methyl ester produces the TNI* that leads to subsequent cleavage of the carboxylic ester group to produce methyl radical. ESR studies in the 1980s showed that the TNI* formed via addition of e_pre_^‒^ to esters undergoes O–C bond cleavage [126]. However, ESR spectral studies have shown that e_pre_^‒^ adds to methyl acetoacetate at 77 K in homogeneous aqueous LiCl glass to form a TNI* that undergoes protonation from the surrounding solvent [127]. Thus, it can be expected that e_pre_^‒^ can cause bond scission via DEA of the TNI*, as shown in Scheme 4. However, ESR studies indicated that the TNI* formed at 77 K, for example, via radiation-produced e_pre_^‒^ addition to thymidine (Thd) or to the nucleotide, 5′-TMP, in homogeneous aqueous LiCl glass, did not lead to bond breakage [30,31,38,39,57,58].

Employing femtosecond pump–probe laser spectroscopy at room temperature in dilute aqueous solution, Lu and co-workers proposed that the TNI* produced via addition of e_pre_^‒^ to selected nucleotides could lead to the cleavage of the sugar-phosphate bond (Scheme 4) [4,101]. However, contrary to this proposal, recent works [43,74,75] employing picosecond pulse radiolysis have clearly and unambiguously established that TNI* formed via addition of e_pre_^‒^ to nucleotides do not cause bond cleavage in the sugar-phosphate backbone in water at room temperature, supporting the above-mentioned ESR spectral results. We summarize our motivation, approach, and important results to resolve this very important controversy in the following section.

As was shown above, LEEs (e_qf_^−^) successively lose energy to become thermalized electrons (e_th_^−^) in a polar medium (e.g., water); in this process, LEEs undergo multistep solvation prior to their complete localization as e_sol_^−^ (Reaction R3, Scheme 1). The transition from e_qf_^‒^ to e_sol_^‒^ is accompanied with the appearance of a strong optical absorption as the electron acquires a stable quantum state. This was established by time-resolved techniques, typically using a short pulse of high-energy electrons or a laser beam [43,74,75]. From the viewpoint of the action of LEEs, it is appropriate to suggest that a thorough understanding of the role played by short-lived non-equilibrated electrons would lead to a clearer picture of the basic mechanisms underlying the biological consequences of radiation. Therefore, a detailed knowledge of electron attachment to DNA/RNA in solution leading to the formation of the TNI* and the subsequent pathways of reactions that the TNI* undergoes (Scheme 4) is of fundamental importance [4,8,29,30,31,38,39,43,45,57,58,59,73,74,75,96,97,98,99,100,101,102,103,104,105,106,107,108,109,120,121]. However, these studies, even at a monomeric DNA-subunit (nucleosides, nucleotides) level, were lacking [43,74,75]. This may be due to challenges encountered in femtosecond laser spectroscopic investigations on the formation of TNI* and its reaction channels. In contrast, the accelerator technique delivers a high-energy electron pulse to the solvent, and hence generates LEEs in accord with those in radiation biology and allows us to investigate the chemistry induced by radiation-produced electrons in liquids.

#### 2.2.2. Picosecond Pulse Radiolysis Measurement of the Initial Yield of Formation of e_sol_^‒^ in a Solution Leads to Study the Reaction of e_pre_^‒^ with Solute

The rationale of this approach is that the interaction of e_pre_^‒^ with nucleobases can be investigated by measuring the initial yield of formation of e_sol_^‒^. e_sol_^‒^ displays a broad absorption band showing a maximum at 715 nm with a relatively high extinction coefficient under ambient conditions and thus can be detected with precision [43,74,75].

#### 2.2.3. Reactivity of e_pre_^‒^ with Nucleobases (X), Nucleosides, and 5′-Nucleotides (XMP) in Water

**Scheme 5:**
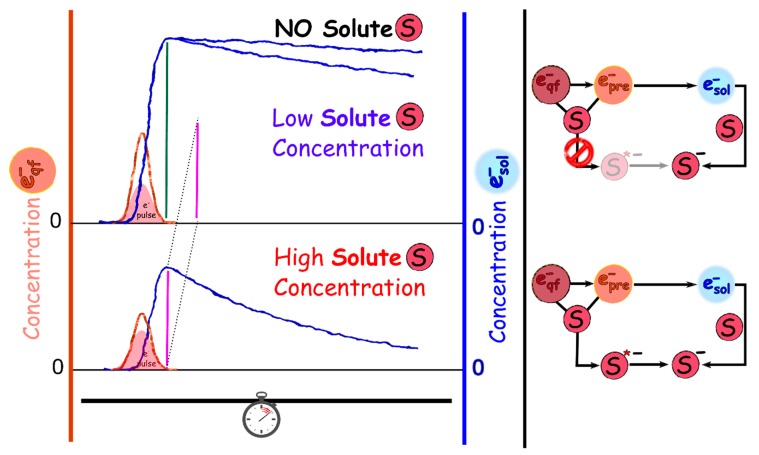
Reaction of e_qf_^−^ and e_pre_^−^ with a solute can be investigated by measuring the initial yield of formation of e_sol_^‒^ at a picosecond timescale.

To test the above-mentioned approach, picosecond (≤ 7 ps) electron pulse (7 MeV) radiolysis coupled with UV-visible (UV-Vis) transient absorption spectroscopy was initially employed to explore the reactivity of e_pre_^‒^ with nucleobases (X), nucleosides, and 5′-nucleotides (XMP) in aqueous solution. It is evident from Scheme 5 that when the precursor of e_sol_^‒^ reacts with nucleobase molecules in competition to its solvation, the yield of formation of e_sol_^‒^ in solutions of nucleobases/nucleotides will decrease in comparison to that of e_sol_^‒^ in water. The laser-triggered continuous probe light of the pulse radiolysis system has the advantage that it covers a broad spectral range from 380 to 1500 nm. This leads to the determination of yields and transient spectra of the resulting intermediates using the electron pulse [43,74,75].

Neither e_pre_^‒^ nor e_sol_^‒^ can induce direct dissociation of the DNA nucleobases via the DEA pathway in aqueous solution: Results obtained employing the ≤ 7 ps pulse radiolysis technique have established that e^‒^ scavenging by DNA nucleobases is not very efficient at moderate DNA nucleobase concentrations (≤ 50 mM) [74], especially, capture of e^‒^ by the purine nucleobases, G and A, does not occur. As a nucleotide has higher intrinsic solubility than its nucleobase, investigation of the trapping of e_pre_^‒^ and of e_sol_^‒^ by a nucleotide became more effective than trapping of these species by the corresponding nucleobase [74]. Pyrimidine nucleobases are found to be more effective electron scavengers, with a decreasing reactivity order of T > C > A > G [74]; these results agree nicely with theoretically calculated values of adiabatic electron affinities of fully hydrated (solvated) nucleobases employing various theoretical methods including density functional theory (DFT) [8,73,121,130,131].

**Figure 5:**
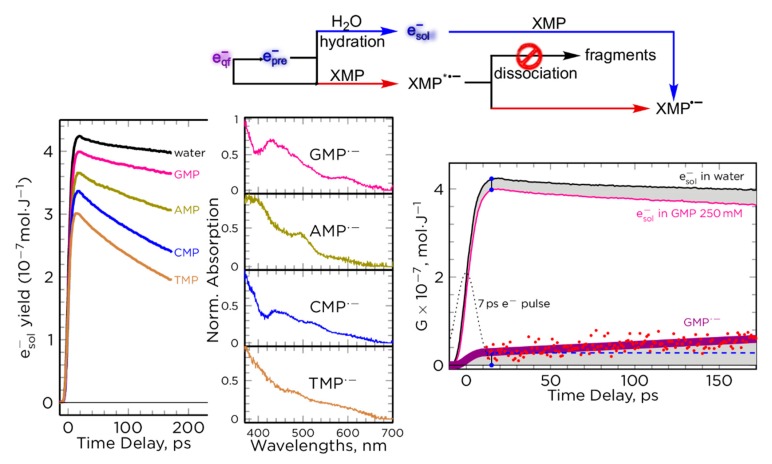
Reactivity of e_qf_^−^ and/or e_pre_^‒^ toward nucleobases and nucleotides in aqueous solution. Right: Yield of the solvated electron versus time and the absorption spectra obtained in addition to that of the e_sol_^‒^ just after the electron pulse. Left: The kinetics of electron and various nucleotides in solution at ambient temperature. Upper part: Mechanism of electron scavenging in solution by various nucleotides also at ambient temperature [74]. Reprinted with permission from [74]. Copyright 2017 American Association for the Advancement of Science.

Thus, these results clearly disagree with those obtained by using femtosecond laser spectroscopy [4,101,102]. However, they agree in part with those obtained in the 1970s by Hunt and coworkers [119]. They had pointed out that the occurrence of effective collisions of e^‒^ with amino acids and mononucleotides depends on the solute concentration and solution pH. Employing the embedded image trapping technique, the spectra of TNI*, i.e., nucleotide anion radicals (XMP^•−^*), formed via addition of e_pre_^‒^ to the nucleotides were observed at 7 ps for the first time (Figure 5). Comparison of the initial electron formation yield with the quantity of electrons scavenged during their hydration suggests that the dissociation pathway of TNI*s (either X^•−^* or XMP^•−^*) and of TNIs (either X^•−^ or XMP^•−^, here the transient negative anion is formed via addition of e_sol_^‒^ to the nucleobase or its corresponding nucleotide) do not occur in the conditions of these experiments (Figure 5).

Thus, these time-resolved studies indicate that neither e_pre_^‒^ nor e_sol_^‒^ can induce direct dissociation of the DNA nucleobases via the DEA pathway (Figure 5). These studies provided the estimated values of rate constants of the reactions of e_pre_^‒^ with nucleotides as: ~5 × 10^12^ M^−1^s^−1^ for thymidine 5′-monophosphate (TMP), 4 × 10^12^ M^−1^s^−1^ for cytidine 5′-monophosphate (CMP), 3 × 10^12^ M^−1^ s^−1^ for adenosine 5′-monophosphate (AMP), and 0.6 × 10^12^ M^−1^s^−1^ for guanosine-5′-monophosphate, GMP. These rate constant values are very important quantities for theoretical calculations to model the reactions of e_pre_^‒^ and e_sol_^‒^ with DNA/RNA [74].

#### 2.2.4. The Reactivity of Quasi-Free Electrons Towards Nucleoside in DEG

In water, trapping and solvation of LEEs (e_qf_^−^) is fast (<100 fs) under ambient conditions (Section 1 and Section 2.2.1–Section 2.2.3). Therefore, investigation of the complete time resolution of the e^‒^ solvation versus its attachment processes is prevented due to the pulse width (5–7 ps) of the current high-energy electron pulse radiolysis system [43,74,75]. Relaxation of the electron (from e_qf_^‒^ to e_pre_^‒^ and ultimately to e_sol_^‒^ (Reaction R3, Scheme 1)) can be viewed as a multistep transition from the delocalized conduction band with *p*-like excited states to *s*-like ground states [4,9,75,130]. Nevertheless, the experimental characterization of the specific state of the electron that is required for the DEA processes remained elusive [75]. Furthermore, formation of the excited states of DNA anion radicals via electron attachment has been suggested [4,8,29,30,31,38,39,43,45,57,58,59,73,74,75,96,97,98,99,100,101,102,103,104,105,106,107,108,109,120,121], but has never been observed [74,75]. In order to achieve these goals, ps pulse radiolysis studies were carried out in diethylene glycol (DEG) for the following reasons: (a) ps Pulse radiolysis in the 1970s and 1980s established that the time of solvation of electrons in alcohols is of the order of several ps [75,132,133]. (b) Electron solvation events have been found to be relatively slow in diethylene glycol (DEG); these multistep events occur in approximately tens of picoseconds. In contrast to water, time resolution of ps pulse radiolysis in DEG provides the opportunity to follow the kinetics of both of e_pre_^−^ and e_sol_^−^ as well as to distinguish between the reactivity of e_qf_^−^ and e_pre_^−^. (c) DEG’s dielectric constant value (ε_r_ = 31.69) has been found to be closer to that of a biological cell than that of water. (d) The native double-stranded structure of DNA and its biological activity can be retained in DEG [75,134], and (e) ribothymidine (rT), a DNA/RNA model, can be sufficiently dissolved (up to 0.5 M) in DEG to scavenge radiation-produced electrons within several ps. Thus, ps pulse radiolysis studies of various concentrations (0 to 5 M) of rT solutions led to the direct observation of the key transient species (e_pre_^‒^, e_sol_^‒^, and TNI* of rT^•‒^*). Furthermore, these studies concluded that within the timescale of the electron pulse < 7 ps, e_qf_^‒^ led to two distinctly localized electron-solvent configuration states-one in the infrared region (as a *p*-like state) and the other in the visible region, which is assigned to a vibrational hot ground state that gradually relaxes to form a solvated electron, e_sol_^‒^ [75]. These results showed that presence of rT does not affect significantly the electron solvation process in DEG; also, rT does not react with e_pre_^‒^ on the time scale of hundreds of ps. Instead, a substantial decrease of the initial near-infrared absorbance was observed that correlated exponentially with increasing rT concentrations, showing an effective reaction was taking place between e_qf_^‒^ and rT.

**Figure 6:**
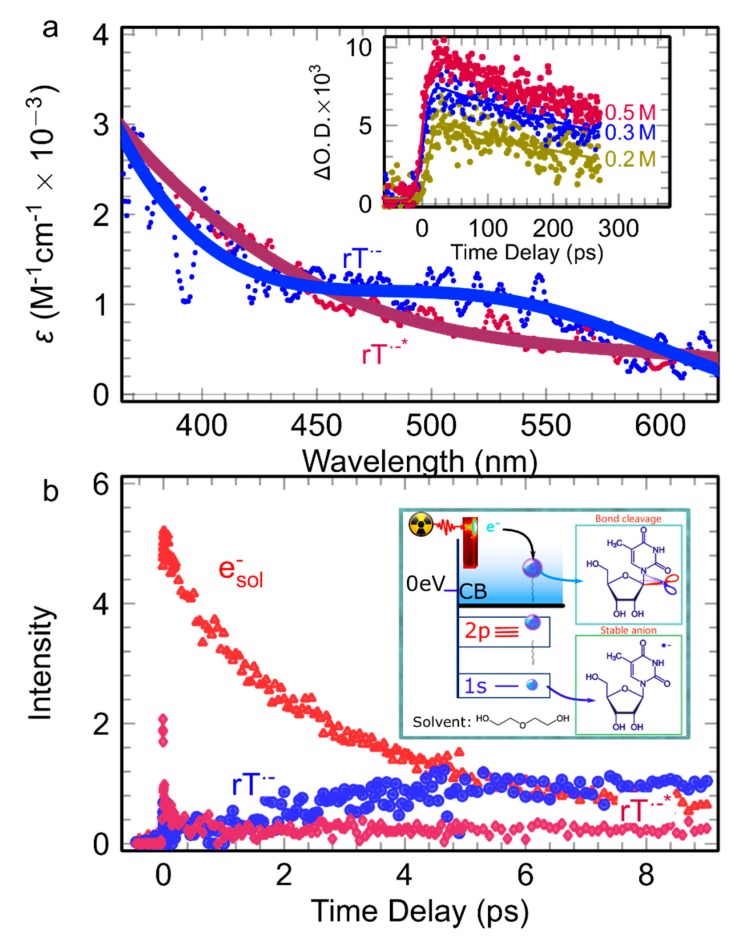
Observation of dissociative e_qf_^‒^ attachment to nucleoside via excited anion radical in solution. (**a**) Absorption spectra of the anion radical at ground (blue) and excited state (red). Inset: decay of the excited state observed at different concentration. (**b**) Kinetics of the different species showing the fast decay of rT^•‒^* and the formation of rT^•‒^ by solvated electron. Inset: Scheme showing the reaction of quasi-free electron forming rT^•‒^* in competition to the electron solvation process [75]. Reprinted with permission from [75]. Copyright 2019 Nature Springer.

The spectrum of rT^•‒^* observed in the ps timescale has been fully characterized [75] (Figure 6), and this spectrum is found to be different from the spectrum of the stable anion radical, rT^•‒^, observed on the microsecond timescale in dilute DEG solutions at room temperature (Figure 6). These time-resolved results presented in Figure 6 in combination with DFT calculations established that (a) rT^•‒^* can be attributed to the excited state πσ*-MO of the anion radical, and (b) dissociation of rT^•−^* can occur via gradual relaxation of the structure by bond elongation leading to a barrier-free N1–C1′ glycosidic bond cleavage through DEA (Scheme 4). These results further imply the generation of biomolecular damage does not necessarily require electrons carrying kinetic energy, i.e., damage to the TNI* (i.e., excited rT anion radical, rT^•−^*) occurs via dissociative electron transfer only by e_qf_^−^ [75].

## 3. Conclusions

The results presented in this review point out that pulse radiolysis is an effective tool to characterize the species (e.g., H_2_O^•+^, TNI*, e_qf_^‒^, e_pre_^‒^, etc.) and to investigate their reactions that are involved in the relevant physicochemical stage of radiation-mediated biomolecular damage by ionizing radiation in solution under ambient conditions.

Two types of processes were investigated in the bulk phase: H_2_O^•+^ mediated hole transfer and excess electron mediated dissociative electron attachment. In the experiments that were carried out using highly concentrated UMP solutions, the UMP^•+^ is generated with a hole distributed over different sites by direct-type effects. The phosphate-sugar hole relaxation dynamics are characterized at a single nucleotide level, and it is clearly observed that the phosphate radical is repaired by sugar via charge and spin transfer. In parallel experiments, 6 M H_3_PO_4_ solutions containing DNA-bases were used to model the phosphate-to-base hole transfer in the absence of a sugar moiety. The excess electron attachment to nucleosides/tides has also been investigated. By observing the decrease of the initial yield of the e_sol_^‒^, the reactivity of e_pre_^‒^ towards DNA has been observed to follow the order of T > C > G >A, and the TNI* does not lead to bond breakage via DEA in liquid water. However, in similar experiments employing DEG solutions instead of water in which the electron relaxation can be resolved, the ps pulse radiolysis results along with DFT calculations showed unequivocally that only e_qf_^‒^ and neither e_pre_^‒^ nor e_sol_^‒^ is able to dissociate the N1–C1′ glycosidic bond in nucleosides via an excited state of the anion radical or TNI*. Recent molecular dynamics studies of the solvation of radiation-produced electrons showed that once LEEs are fully solvated (Reaction R3, Scheme 1), their reactivity is governed and limited by diffusion and hence the eventual damage due to radiation-produced electrons will be confined within the size of a cell [135]. Thus, if radiation sensitizers can be targeted only to the hypoxic tumor cells, better therapeutic efficacy will be achieved. Thus, the ps pulse radiolysis results are not only benchmarks for future theoretical calculations and experimental studies to elucidate radiation-mediated biomolecular damage, but they can also be employed to study hypoxic cell radiosensitizers.

## Figures and Tables

**Table 1:** Rate constants of the bimolecular reactions of DNA bases with H_2_PO_4_^•^ in 6 M H_3_PO_4_ along with standard redox potentials of nucleotide base cation radicals [44].

DNA Bases	*k* (H_2_PO_4_^•^) L mol^−1^s^−1^	E^0^/V (dB^•+^/dB)
G	6.9 × 10^8^	1.47
A	2.4 × 10^8^	1.94
T	1.1 × 10^9^	2.09
C	<5 × 10^7^	2.12
